# Infidelity Analysis of Digital Counter-Diabatic Driving in Simple Two-Qubit System

**DOI:** 10.3390/e26100877

**Published:** 2024-10-19

**Authors:** Ouyang Lei

**Affiliations:** Department of Physics, Shanghai University, Shanghai 200444, China; reo@shu.edu.cn

**Keywords:** counter-diabatic driving, Suzuki–Trotter decomposition, error analysis, optimization

## Abstract

Digitized counter-diabatic (CD) optimization algorithms have been proposed and extensively studied to enhance performance in quantum computing by accelerating adiabatic processes while minimizing energy transitions. While adding approximate counter-diabatic terms can initially introduce adiabatic errors that decrease over time, Trotter errors from decomposition approximation persist. On the other hand, increasing the high-order nested commutators for CD terms may improve adiabatic errors but could also introduce additional Trotter errors. In this article, we examine the two-qubit model to explore the interplay between approximate CD, adiabatic errors, Trotter errors, coefficients, and commutators. Through these analyses, we aim to gain insights into optimizing these factors for better fidelity, a shallower circuit depth, and a reduced gate number in near-term gate-based quantum computing.

## 1. Introduction

The adiabatic theorem ensures that a quantum system remains in its eigenstate if the time-dependent parameters in the Hamiltonian evolve slowly enough. This principle gives rise to an analog paradigm for quantum computing, which aims to minimize functions encoded in spin-problem Hamiltonians [1,2]. In adiabatic quantum computing [3,4], the system is initially prepared in a superposition state as the eigenstate of a transverse-field Hamiltonian. Gradually, the transverse field is turned off, while the problem Hamiltonian is turned on synchronously. This process ideally leads the system to its ground state, which encodes the classical solution to the problem. However, if the process is non-adiabatic, the final state will deviate from the ground state, resulting in inaccurate solutions that are difficult to verify. The induced adiabatic errors are usually inevitable because the coherence time of the quantum system is limited [5], necessitating a shorter operation time to preserve quantum coherence. This insight has inspired the development of a quantum annealer that solves the quadratic unconstrained binary optimization (QUBO) problem by exploiting the principles of adiabatic theorem and quantum tunneling with thousands of noisy flux qubits [6,7]. Although quantum annealers have shown remarkable advantages in scalability, there are still some fundamental limitations. In addition to the coherence time, the most critical issues are the feasible interaction types and qubit connectivity [8]. More specifically, one cannot use the SWAP operation to embed the problem without a gate model. Therefore, minor embedding must be addressed as a preliminary step to map the problem to the topology graph of the annealer.

To circumvent this issue, the paradigm of digital adiabatic quantum computing has been proposed [9], offering a more flexible and equivalent approach. It has been demonstrated in a superconducting circuit quantum computer with pioneering experimental technology. Using Suzuki–Trotter decomposition [10,11,12,13], it replaces continuous evolution with blocks of quantum gates that approximate the quantum dynamics during discrete timesteps. This allows for the simulation of quantum annealing with longer annealing times while preserving quantum coherence. This paradigm is also related to variational algorithms, such as the quantum approximate optimization algorithm (QAOA) [14,15,16,17,18], which minimizes energy by optimizing the digitized annealing schedule. Additionally, adiabatic errors can be suppressed by introducing auxiliary interactions to realize counter-diabatic driving [19,20,21,22,23]. Although such terms are not implementable in state-of-the-art quantum annealers, they can be effectively realized in digital adiabatic quantum computing and variational algorithms [24,25]. However, the decomposition introduces a new source of infidelity known as Trotter errors [26,27]. Specifically, canceling adiabatic errors through counter-diabatic driving can increase Trotter errors, suggesting an interplay between diabatic and Trotter errors. It is, therefore, natural to explore whether counter-diabatic driving can be further optimized to compensate not only for energy excitations but also for the infidelity induced via Trotter decomposition.

In this article, we present an infidelity analysis of digital counter-diabatic quantum computing in a two-qubit system. As a minimal model, it provides an analytical approach to studying infidelity induced via both adiabatic errors and Trotter errors, allowing for extensions to larger models. Using nested commutators [22], we derive explicit time-dependent auxiliary terms that cancel energy excitations induced via a non-adiabatic annealing schedule. We calculate the coefficients of the first- and second-order nested commutators, evaluating the infidelity induced via Trotterizations. Additionally, we propose optimized coefficients to mitigate Trotter errors, allowing a scaling-up to multiple qubit problems. By examining the interplay between adiabatic errors and Trotter errors, we gain insight into optimizing the performance of digital adiabatic quantum computing, achieving better fidelity and a shallower circuit depth in near-term gate-based quantum computing.

## 2. Digitized Counter-Diabatic Driving

Adiabatic quantum computing can be characterized by a time-dependent Hamiltonian:

(1) $$H_{0}\left(t\right)=\left[1-\lambda \left(t\right)\right]H_{i}+\lambda \left(t\right)H_{f},$$

where $H_{i}$ and $H_{f}$ are the initial Hamiltonian with a trivial ground state and the final Hamiltonian to be solved, respectively. According to the adiabatic theorem, the quantum system is expected to remain in the instantaneous eigenstate of $H_{0}\left(\lambda \right)$ if the parameter is tuned slowly enough. This process results in the ground state of $H_{f}$ if λ(t) satisfies the boundary conditions λ(0)=0 and λ(T)=1. In practice, the operation time required to maintain adiabatic criteria is longer than the coherence time of the quantum device. Consequently, the actual operation time, known as the annealing time in a quantum annealer, is reduced, inducing unwanted energy excitations due to the diabatic effect. One solution is to find an optimal annealing schedule, whose digitized version is equivalent to QAOA. A parallel approach, called counter-diabatic (CD) driving, suppresses energy excitations by adding an auxiliary term, $H_{CD}$, to the total Hamiltonian while keeping λ(t) fixed. For a many-body Hamiltonian, the nested commutator method provides the CD terms in a series of *l*-order as follows:

(2) $$\begin{array}{ccc} H_{CD} & = & \dot{\lambda }A_{\lambda }, \end{array}$$

(3) $$\begin{array}{ccc} A_{\lambda }^{\left(l\right)} & = & i\sum_{k=1}^{l}\alpha_{k}\underset{2k-1}{\underset{︸}{[H_{0},[H_{0},\ldots ,[H_{0}}},\partial_{\lambda }H_{0}]\ldots ]], \end{array}$$

Here, the index *k* refers to the *k*-th nested commutator. Indeed, the exact gauge potential for diabatic transitions in the l→∞ limit. The coefficients $\alpha_{k}$ can be derived by minimizing the effective action:

(4) $$\begin{array}{ccc} S & = & \mathrm{Tr}\left(G_{l}^{2}\right),\\ G_{l} & = & \partial_{\lambda }H_{0}-i\left[H_{0},A_{\lambda }^{\left(l\right)}\right]. \end{array}$$

Thus, one has the propagator

(5) $$\begin{array}{c} U\left(t,0\right)=T\exp\left[-i\int_{0}^{t}\left(H_{0}\left(t^{\prime }\right)+H_{CD}\left(t^{\prime }\right)\right)dt^{\prime }\right], \end{array}$$

that provides the wave function |Ψ(t)〉=U(t,0)|Ψ(0)〉 at any arbitrary time t∈[0,T]. Note that the gauge potential $A_{\lambda }^{\left(l\right)}$ is not perfect when *l* is finite, i.e., an adiabatic error still exists with a truncation of the gauge potential, causing the wave function |Ψ(t)〉 to deviate from the instantaneous eigenstate $|\tilde{\Psi }\left(t\right)\rangle$.

As we can see, the gauge potential is a series of commutators, consisting of interaction terms that are not implementable in state-of-the-art quantum annealers. However, this is no longer a problem in digital adiabatic quantum computing, as the propagator U(T,0) can be simulated in gate-model quantum computers and Suzuki–Trotter decomposition:

(6) $${\hat{U}}_{\mathrm{dig}}\left(T,0\right)=\prod_{n=1}^{M}e^{-iH_{0}\left(n\delta t\right)\delta t}e^{-iH_{CD}\left(n\delta t\right)\delta t},$$

where *M* is the number of Trotter steps of length δt. With a finite *M*, the circuit comprises *M* blocks of the same structure, leading to Trotter errors that scale as $𝒪(\delta t^{2})$ [27]. It is important to note that the CD terms introduce additional Trotter errors due to their non-commutation with both the initial Hamiltonian, $H_{i}$, and the final Hamiltonian, $H_{f}$.

We analyze the infidelity, defined as ϵ=1−F, where $F\left(t\right)=|\langle \tilde{\Psi }\left(t\right)|\Psi_{\mathrm{dig}}\left(t\right)\rangle {|}^{2}$ is the probability overlap between the wave function and the instantaneous eigenstate. Infidelity arises from both adiabatic errors due to imperfect CD terms and Trotter errors from digitalization. There is a trade-off between these errors, as adiabatic errors can be reduced by increasing order *l* of the gauge potential $A_{\lambda }^{\left(l\right)}$, which simultaneously introduces extra Trotter errors from nested commutators. Meanwhile, a higher order of Suzuki–Trotter decomposition reduces the Trotter errors at the cost of more operators, resulting in a deeper circuit and larger gate numbers. By analyzing nested commutators in approximate CD terms and infidelity, it is possible to refine these variables to enhance fidelity without increasing circuit depth.

## 3. Two-Qubit System

We consider the two-qubit Hamiltonian $H_{0}\left(\lambda \right)$ that consists of the initial and final Hamiltonians as follows:

(7) $$\begin{array}{c} \begin{array}{cc} H_{i} & =-2J\sigma_{1}^{z}\sigma_{2}^{z}-h\left(\sigma_{1}^{z}+\sigma_{2}^{z}\right),\\ H_{f} & =-2J\sigma_{1}^{z}\sigma_{2}^{z}-h\left(\sigma_{1}^{z}+\sigma_{2}^{z}\right)+2h\left(\sigma_{1}^{x}+\sigma_{2}^{x}\right), \end{array} \end{array}$$

where the spin-0 state $(|↑↓\rangle -\left|↓↑\rangle \right)/\sqrt{2}$ is decoupled from the Hilbert space, effectively resembling a three-level system. Accordingly, we have the following annealing schedule:

(8) $$\lambda \left(t\right)=\sin^{2}\left[\left(\pi /2\right)\sin^{2}\left(\pi t/2T\right)\right],$$

satisfying the boundary conditions λ(0)=0 and λ(T)=1. The first- and second-order derivatives at t=0 and t=T are zero, ensuring smooth quantum annealing. Thus, one can calculate the gauge potential of arbitrary order *l*. For instance, the first-order expansion of the nested commutator leads to the following:

(9) $$A_{\lambda }^{\left(1\right)}=8\alpha_{1}Jh\left(\sigma_{1}^{y}\sigma_{2}^{z}+\sigma_{1}^{z}\sigma_{2}^{y}\right)+4\alpha_{1}h^{2}\left(\sigma_{1}^{y}+\sigma_{2}^{y}\right),$$

where the coefficient is given as follows:

(10) $$\alpha_{1}=-\frac{J^{2}+h^{2}/4}{{\left(4J^{2}+h^{2}\right)}^{2}+{\left(4Jh\right)}^{2}+{\left(2\lambda h^{2}\right)}^{2}+{\left(8J\lambda h\right)}^{2}}.$$

Therefore, $A_{\lambda }^{\left(1\right)}$ provides a pool of CD operators as $\left\{\sigma_{1}^{y}\sigma_{2}^{z},\sigma_{1}^{z}\sigma_{2}^{y},\sigma_{1}^{y},\sigma_{2}^{y}\right\}$. The second-order gauge potential $A_{\lambda }^{\left(2\right)}$ is also explicitly solvable, introducing two more operators, $\left\{\sigma_{1}^{x}\sigma_{2}^{y},\sigma_{1}^{y}\sigma_{2}^{x}\right\}$, to the pool. It is worthwhile to emphasize that the algebra forbids the existence of other types of CD operators with higher orders of the nested commutator. Meanwhile, $A_{\lambda }^{\left(2\right)}$ is indeed the *exact* gauge potential since there are only two excitation frequencies. On the other hand, obtaining an analytical solution becomes increasingly challenging as the size of the systems expands. The CD terms of the two-qubit system can be readily obtained at a truncation l=2. Thus, it can be considered an illustrative example to elucidate the impact of CD terms on adiabatic errors, as well as Trotter errors.

**Figure 1:**
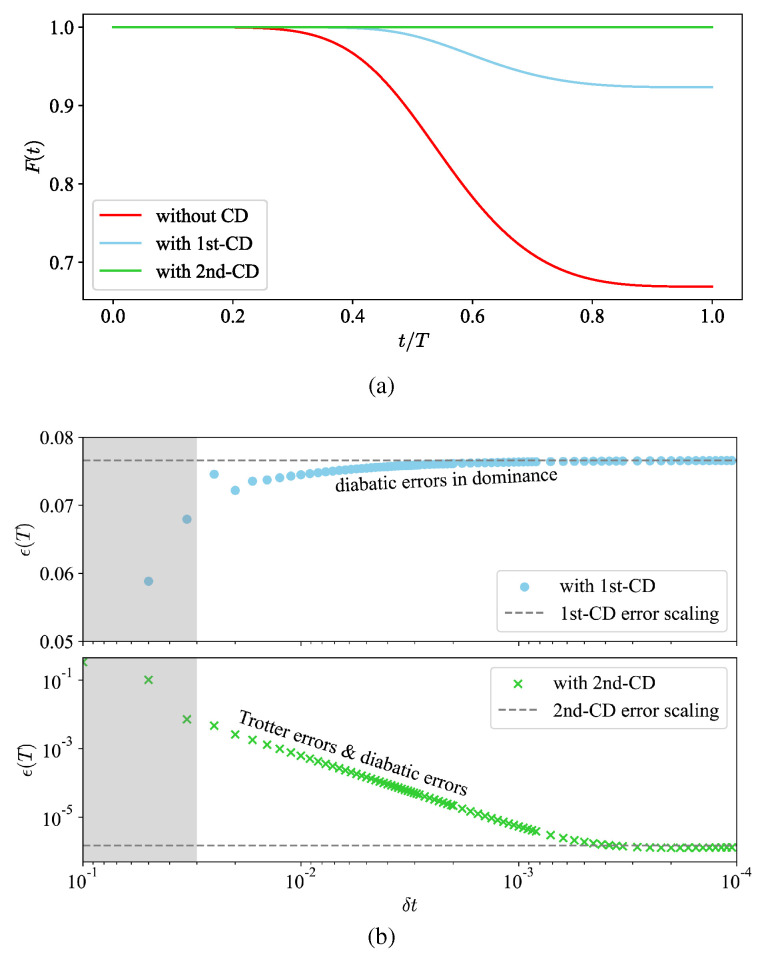
(**a**) Fidelity F(t) between the evolving state and the instantaneous ground state in the two-qubit system for the original Hamiltonian and CD driving protocols with the first and second orders. (**b**) Final infidelity of digitized approximate simulations, ϵ(T)=1−F(T), against the time interval δt of each step. In the shaded regions, the Trotter product formula is non-convergent. The dashed lines show error scalings, about $7.7\times 10^{-2}$ and $1.5\times 10^{-6}$ separately for the first- and second-order driving. The former is mainly attributed to adiabatic errors, while the latter stems from the accuracy limitations of simulation computing.

For the numerical simulation, the QuTiP library [28,29] is utilized for the state vector multiplication in Python code (v.3.9.7). We set the annealing time to T=0.1, the exchange energy to J=1, and the local bias to h=2. As shown in Figure 1a, the conventional Hamiltonian $H_{0}\left(\lambda \right)$ describes a non-adiabatic process, resulting in a fidelity of around F=0.67. With the first- and second-order nested commutators for the CD terms, the fidelity increases to around F=0.92 and F=1, respectively.

The annealing process can be digitized for implementation in gate-model quantum computers. By varying the time interval δt, we observe both diabatic and Trotter errors, as shown in Figure 1b. Adiabatic errors primarily contribute to the infidelity in the l=1 CD protocol, while they vanish in the l=2 CD protocol. Meanwhile, Trotter error-induced infidelity is linearly suppressed on a logarithmic scale as δt decreases. Thus, we analyze the infidelity using the Fubini–Study angle [30], aiming to enhance fidelity through optimizing the digitized CD terms with insights gained explicitly from Taylor expansion and the Baker–Campbell–Hausdorff (BCH) formula.

### 3.1. Improving Adiabatic Errors with l=1 CD

**Figure 2:**
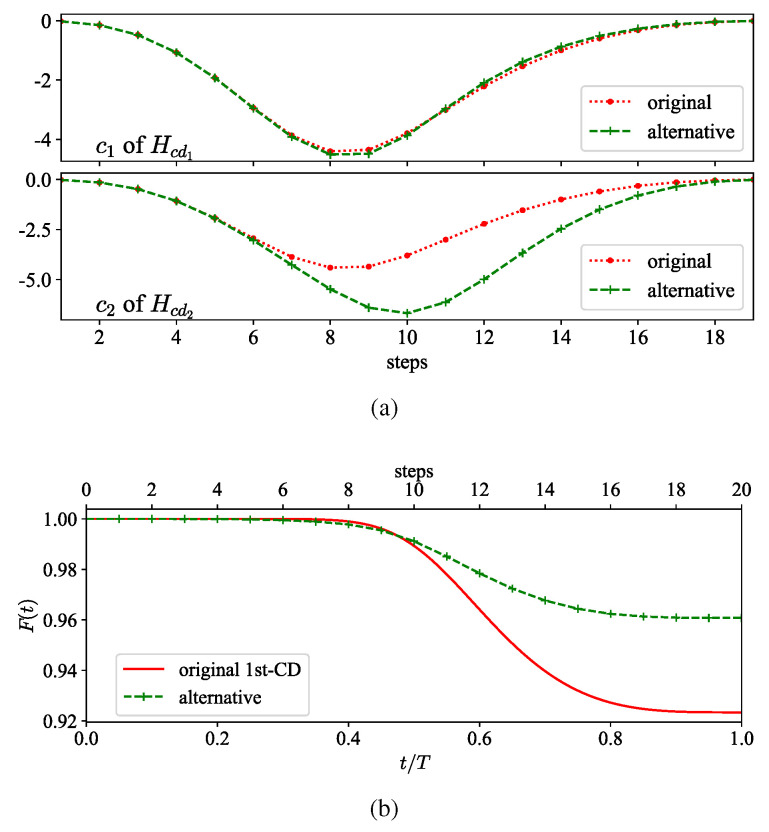
(**a**) Original coefficients and the alternative under Trotter steps M=20. The alternative coefficients are selected from the ones associated with $H_{CD}^{\left(1\right)}$ and $H_{CD}^{\left(2\right)}$ in the second-order CD terms. (**b**) Fidelity F(t) of the first-order CD driving with the original coefficients and the alternative.

We first consider improving the performance of digital counter-diabatic computing with a l=1 gauge potential, which corresponds to a shallower quantum circuit. As shown in Figure 1b, adiabatic errors are dominant when the order of the nested commutator is set to l=1. Therefore, it is reasonable to focus solely on reducing the adiabatic errors without increasing the circuit depth while postponing efforts to reduce the Trotter errors. The operator pool of $A_{\lambda }^{\left(1\right)}$ can be divided into two sub-Hamiltonians:

(11) $$\begin{array}{ccc} H_{CD}^{\left(1\right)} & = & \sigma_{1}^{y}\sigma_{2}^{z}+\sigma_{1}^{z}\sigma_{2}^{y}, \end{array}$$

(12) $$\begin{array}{ccc} H_{CD}^{\left(2\right)} & = & \sigma_{1}^{y}+\sigma_{2}^{y}, \end{array}$$

resulting in the digitized CD propagator

(13) $$U_{CD}\left[n\delta t,\left(n-1\right)\delta t\right]=\prod_{j=1}^{2}e^{-ic_{j}\left(n\delta t\right)H_{CD}^{\left(j\right)}\delta t},$$

where $c_{j}\left(n\delta t\right)$ are the corresponding coefficients at t=nδt. Note that adiabatic errors can be further canceled by increasing the order of the nested commutator. We observe that both local and two-body operators of $H_{CD}^{\left(1\right)}$ and $H_{CD}^{\left(2\right)}$ also appear in the operator pool of the gauge potential $A_{\lambda }^{\left(2\right)}$. Hence, we incorporate the corresponding coefficients from the l=2 CD terms to improve fidelity without increasing the circuit depth by introducing more types of CD operators. Figure 2a serves as an example, depicting the variation in the coefficients at M=20 Trotter steps, where the coefficient $c_{1}$ for two-body interaction is slightly adjusted, but the coefficient $c_{2}$ for local rotation is significantly changed. The real-time optimized infidelity between the evolved state of digital CD driving and the instantaneous ground state is illustrated in Figure 2b, reducing the final infidelity to $\epsilon =3.7\times 10^{-2}$.

We attempt to explain the effectiveness by analyzing the geometrical distance between the final state, $|\Psi_{dig}\left(T\right)\rangle$, and the target state, $|\tilde{\Psi }\left(T\right)\rangle$, based on the Fubini–Study metric:

(14) $$\theta_{FS}(|\tilde{\Psi }\left(T\right)\rangle ,|\Psi_{dig}\left(T\right)\rangle )=\arccos|\langle \tilde{\Psi }\left(T\right)|\Psi_{dig}\left(T\right)\rangle |.$$

By applying the triangle inequality, we obtain an inequality that aggregates an error upper bound over *M* steps:

(15) $$\theta_{FS}(|\tilde{\Psi }\left(T\right)\rangle ,|\Psi_{dig}\left(T\right)\rangle )\leq \sum_{n=1}^{M}\Theta_{n},$$

where

(16) $$\Theta_{n}=\arccos|\langle \tilde{\Psi }\left(n\delta t\right)|{\hat{U}}_{dig}\left[n\delta t,\left(n-1\right)\delta t\right]|\tilde{\Psi }\left[\left(n-1\right)\delta t\right]\rangle |.$$

$\Theta_{n}$ characterizes the overlap between the deviated state after evolving a single Trotter step on the instantaneous eigenstate $|\tilde{\Psi }\left[\left(n-1\right)\delta t\right]\rangle$ and the ideal instantaneous eigenstate $|\tilde{\Psi }\left[n\delta t\right]\rangle$. Hence, the smaller $\Theta_{n}$ is, the more ${\hat{U}}_{\mathrm{dig}}$ approximates ideal adiabatic quantum computing.

**Figure 3:**
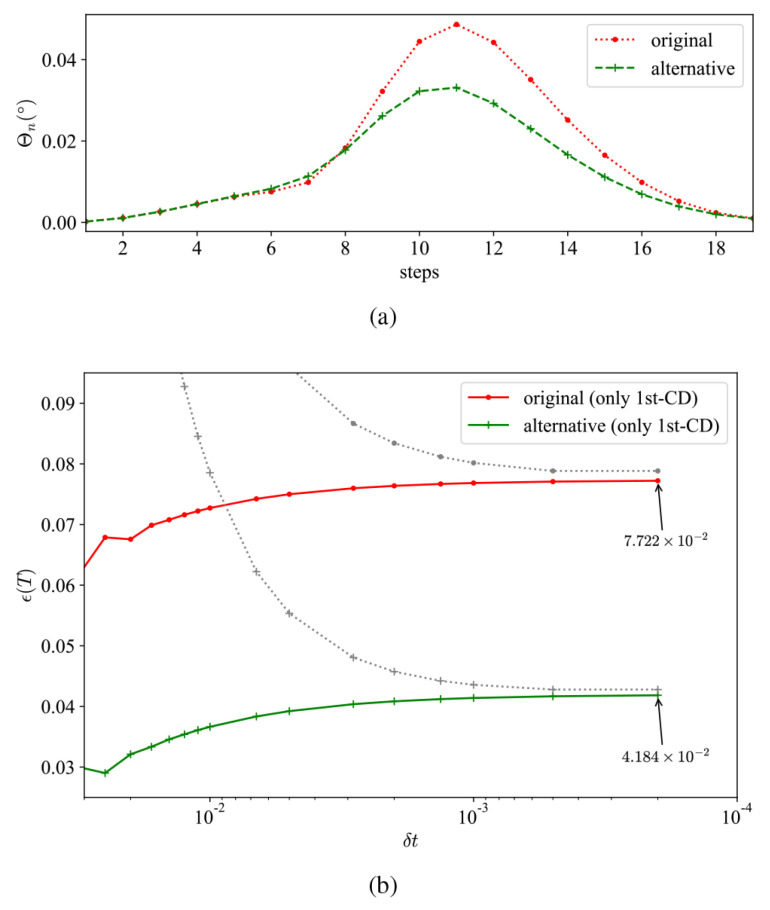
(**a**) The distance between digital simulated dynamics and true dynamics at each step for original and alternative drivings. (**b**) The infidelity of the two drivings against the time interval δt. The gray dotted lines are the error upper-bound derived from Fubini–Study angles.

We illustrate the evolution of $\Theta_{n}$ at M=20 Trotter steps with the standard l=1 CD protocol and its modification in Figure 3a. The angle $\Theta_{n}$, after the coefficients from terms in l=2 are partially combined, is significantly decreased, demonstrating that the performance is improved by steering the state along the instantaneous ground state. Additionally, the elements contributing to the improvement of fidelity are presented in Appendix A.

By merging up the coefficients, we observe a reduction in the upper bound on errors. In Figure 3b, we present the upper bounds for both the conventional l=1 CD protocol and the merged protocol, maintaining the same circuit depth. Summing the reduced Fubini–Study angles, $\Theta_{n}$, results in a looser upper bound due to the nature of triangle inequality. As the number of Trotter steps, *M*, increases, the upper bound will gradually tighten until it aligns with the error scaling.

### 3.2. Improving Trotter Errors with l=2 CD

By increasing the order of the nested commutator, the adiabatic errors is significantly reduced. The two-qubit Hamiltonian with the l=2 CD protocol demonstrates that the evolution closely follows the instantaneous eigenstate of the original Hamiltonian. Therefore, this Hamiltonian serves as an appropriate model for studying Trotter errors and their suppression. It introduces an additional sub-Hamiltonian to the operator pool of $A_{\lambda }^{\left(2\right)}$ as $H_{CD}^{\left(3\right)}=\sigma_{1}^{x}\sigma_{2}^{y}+\sigma_{1}^{y}\sigma_{2}^{x}$, which subsequently gives the digitized CD propagator

(17) $$U_{CD}\left[n\delta t,\left(n-1\right)\delta t\right]=\prod_{j=1}^{3}e^{-ic_{j}\left(n\delta t\right)H_{CD}^{\left(j\right)}\delta t}.$$

As shown in Figure 1b, when the number of Trotter steps is limited, a longer δt causes a significant difference between the Hamiltonians at consecutive time intervals. This results in a noticeable presence of commutators and nested commutators in the higher-order terms of the Trotter decomposition, which introduces Trotter errors. To explain this further, the higher-order terms are derived by applying a Taylor expansion to the exponents. The product of three exponents in one Trotter step can be expressed as in Equation (22), which is related to the well-known BCH formula. When δt→0, the first-order Trotter decomposition is enough to achieve high precision. This means that the effects of higher-order expansions are mainly influenced by δt and the coefficients, while commutators and nested commutators determine the operators that can be classified. Typically, higher-order expansions introduce additional operators beyond the CD operator pool. However, in this system, the commutative relationship between these operators is straightforward: $\left[H_{CD}^{\left(j\right)},H_{CD}^{\left(k\right)}\right]=-2i\varepsilon_{jkl}H_{CD}^{\left(l\right)}$ with j,k,l=1,2,3. Therefore, the commutators revert back to operators already included in the CD operator pool. This means there is a closure of the commutative relationship for the operators in the second-order CD terms.

To replace the commutators in Equation (22) and collect the terms of the same operators, we have

(18) $$\begin{array}{cc} \; & \prod_{j=1}^{3}e^{-ic_{j}H_{CD}^{\left(j\right)}\delta t}\\ \; & =e^{-i\delta t\sum_{j=1}^{3}c_{j}H_{CD}^{\left(j\right)}+\frac{2i}{2}{\left(-i\delta t\right)}^{2}\left\{-c_{1}c_{2}H_{CD}^{\left(3\right)}+c_{1}c_{3}H_{CD}^{\left(2\right)}-c_{2}c_{3}H_{CD}^{\left(1\right)}\right\}+\ldots }\\ \; & =e^{-i\delta t\left\{\left(c_{1}-\delta tc_{2}c_{3}+\ldots \right)H_{CD}^{\left(1\right)}+\left(c_{2}+\delta tc_{1}c_{3}+\ldots \right)H_{CD}^{\left(2\right)}+\left(c_{3}-\delta tc_{1}c_{2}+\ldots \right)H_{CD}^{\left(3\right)}\right\}}\\ \; & =e^{-i\delta t({\tilde{c}}_{1}H_{CD}^{\left(1\right)}+{\tilde{c}}_{2}H_{CD}^{\left(2\right)}+{\tilde{c}}_{3}H_{CD}^{\left(3\right)})}. \end{array}$$

In this context, the combination of coefficients is represented by new coefficients, ${\tilde{c}}_{j}$, resulting in an expression that resembles an effective evolution operator ${\hat{U}}_{CD,discrete}=e^{-i\delta t\left(c_{1}H_{CD}^{\left(1\right)}+c_{2}H_{CD}^{\left(2\right)}+c_{3}H_{CD}^{\left(3\right)}\right)}$. Since contributions from higher-order terms are included, this allows for the possibility of offsetting Trotter errors during the decomposition process by comparing with ${\hat{U}}_{CD,discrete}$. For instance, the coefficients in digitization can be adjusted accordingly. The specifics are as follows: substitute $c_{j}\left(n\delta t\right)$ with ${\tilde{c}}_{j}$, and the coefficients that require modification in the product (18) are renamed as $c_{j}^{*}$, which can be obtained by solving a series of transformation equations. Below are the equations for the second-order approximation.

(19) $$\left\{\begin{array}{cc} c_{1}\left(n\delta t\right) & =c_{1}^{*}-\delta tc_{2}^{*}c_{3}^{*}\\ c_{2}\left(n\delta t\right) & =c_{2}^{*}+\delta tc_{1}^{*}c_{3}^{*}\\ c_{3}\left(n\delta t\right) & =c_{3}^{*}-\delta tc_{1}^{*}c_{2}^{*} \end{array}\right.,$$

and the third-order approximation

(20) $$\left\{\begin{array}{cc} c_{1}\left(n\delta t\right) & =c_{1}^{*}-\delta tc_{2}^{*}c_{3}^{*}-\frac{\delta t^{2}}{3}\left({\left(c_{2}^{*}\right)}^{2}+{\left(c_{3}^{*}\right)}^{2}\right)c_{1}^{*}\\ c_{2}\left(n\delta t\right) & =c_{2}^{*}+\delta tc_{3}^{*}c_{1}^{*}-\frac{\delta t^{2}}{3}\left({\left(c_{1}^{*}\right)}^{2}+{\left(c_{3}^{*}\right)}^{2}\right)c_{2}^{*}\\ c_{3}\left(n\delta t\right) & =c_{3}^{*}-\delta tc_{1}^{*}c_{2}^{*}-\frac{\delta t^{2}}{3}\left({\left(c_{1}^{*}\right)}^{2}+{\left(c_{2}^{*}\right)}^{2}\right)c_{3}^{*} \end{array}\right..$$

Essentially, this procedure aims to approximate ${\hat{U}}_{cd,discrete}$ as closely as possible, ultimately achieving equality when the Taylor expansion is infinite. To balance computational efficiency with accuracy, we utilize the Taylor expansion up to the third order. The number of equations is determined by the number of coefficients, which can be reduced by merging terms of mutually commutative operators. The process of solving these equations must be repeated *M* times to minimize Trotter errors at each Trotter step. The final infidelity can be improved by applying the solutions $c_{j}^{*}$ in chronological order during digitization, which rewrites the optimized time-evolution operator as ${\hat{U}}^{*}\left(0,T\right)=\prod_{n=1}^{M}\prod_{j=1}^{3}e^{-ic_{j}^{*}H_{CD}^{\left(j\right)}\delta t}$.

**Figure 4:**
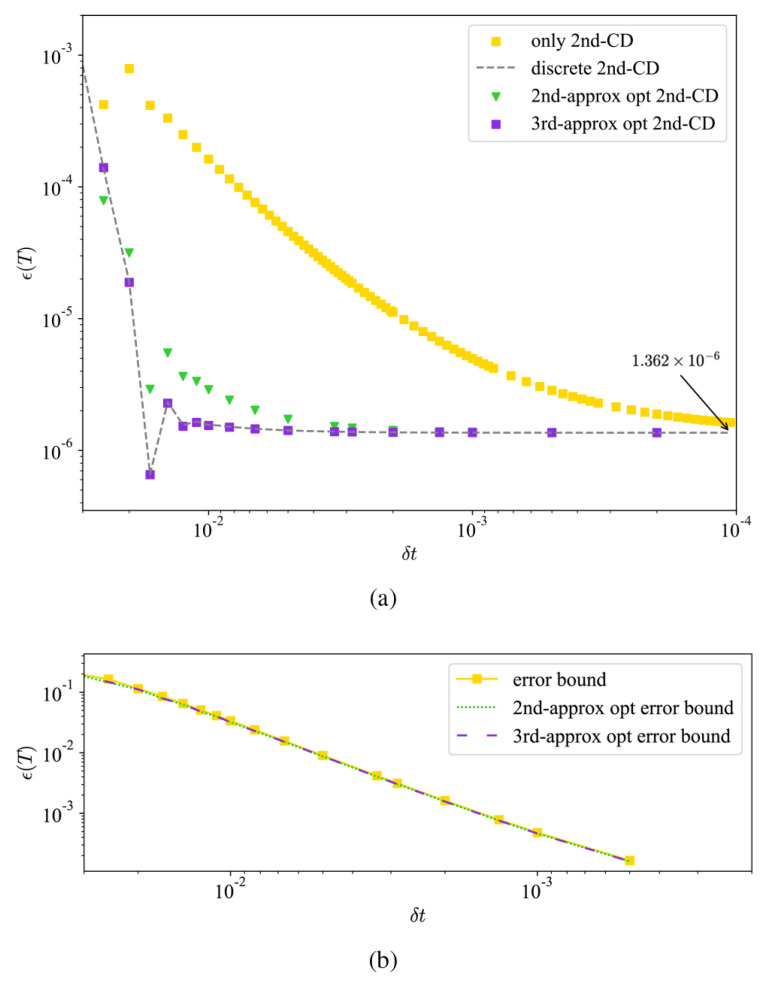
(**a**) The infidelity, ϵ(T), of the second-order CD driving using optimized coefficients in the second- and third-order approximation. The dashed line is the result of the discrete effective operators. All of these drivings converge to an error scaling of $1.362\times 10^{-6}$. (**b**) The error upper-bound derived from Fubini–Study angles for the second-order CD driving with and without the optimization. The figure shows that the optimizations in coefficients are very delicate. Moreover, the upper bounds are much higher than the corresponding infidelity in (**a**), suggesting that l=2 is sufficient for high-accuracy fidelity.

The results, illustrated in Figure 4a, show notable improvements in Trotter errors within the range of 0.02≥δt≥0.001, and they quickly approach the same error scaling within a limited number of total steps, *M*. Based on the property of closure in commutators among the CD operator pool, the optimization of digitization for a two-qubit system can be enhanced by modifying the coefficients at all steps. In general, superior digital dynamics can be achieved for any Hamiltonian of CD terms that possess such a feature.

Additionally, the error upper bounds of different conditions derived from Equation (15) are also contained in Figure 4b, showing that the inclusion of second-order CD terms is sufficient to achieve high-accuracy fidelity.

(21) $$\begin{array}{cc} cos(\Theta_{n}) & =|\langle {\tilde{\psi }}_{n}|e^{-iH_{0}\delta t}\prod_{j=1}^{2}e^{-ic_{j}H_{CD}^{\left(j\right)}\delta t}|{\tilde{\psi }}_{n-1}\rangle |\\ \; & \approx |\langle {\hat{U}}_{0,n}^{†}{\tilde{\psi }}_{n}|\left(I-i\delta t\left\{c_{1}H_{CD}^{\left(1\right)}+c_{2}H_{CD}^{\left(2\right)}\right\}+\frac{1}{2}{\left(-i\delta t\right)}^{2}\left\{{\left(c_{1}H_{CD}^{\left(1\right)}\right)}^{2}+{\left(c_{2}H_{CD}^{\left(2\right)}\right)}^{2}+2c_{1}c_{2}H_{CD}^{\left(1\right)}H_{CD}^{\left(2\right)}\right\}\right)|{\tilde{\psi }}_{n-1}\rangle |\\ \; & =|e^{iE_{0}\delta t}\left(\langle {\tilde{\psi }}_{n}|{\tilde{\psi }}_{n-1}\rangle +\langle {\tilde{\psi }}_{n}|𝒪\left(\delta t\right)|{\tilde{\psi }}_{n-1}\rangle +\langle {\tilde{\psi }}_{n}|𝒪\left(\delta t^{2}\right)|{\tilde{\psi }}_{n-1}\rangle \right)|\\ \; & =|\left(a_{0}+ib_{0}\right)+\delta t\left(a_{1}+ib_{1}\right)+\delta t^{2}\left(a_{2}+ib_{2}\right)| \end{array}$$

(22) $$\begin{array}{cc} \; & \prod_{j=1}^{3}e^{-ic_{j}H_{CD}^{\left(j\right)}\delta t}\\ \; & =I-i\delta t\left\{\sum_{j=1}^{3}c_{j}H_{CD}^{\left(j\right)}\right\}+\frac{1}{2}{\left(-i\delta t\right)}^{2}\left\{\sum_{j=1}^{3}{\left(c_{j}H_{CD}^{\left(j\right)}\right)}^{2}+2c_{1}c_{2}H_{CD}^{\left(1\right)}H_{CD}^{\left(2\right)}+2c_{1}c_{3}H_{CD}^{\left(1\right)}H_{CD}^{\left(3\right)}+2c_{2}c_{3}H_{CD}^{\left(2\right)}H_{CD}^{\left(3\right)}\right\}+\ldots \\ \; & =I-i\delta t\left\{\sum_{j=1}^{3}c_{j}H_{CD}^{\left(j\right)}\right\}+\frac{1}{2}{\left(-i\delta t\right)}^{2}\left\{{\left(\sum_{j=1}^{3}c_{j}H_{CD}^{\left(j\right)}\right)}^{2}+c_{1}c_{2}\left[H_{CD}^{\left(1\right)},H_{CD}^{\left(2\right)}\right]+c_{1}c_{3}\left[H_{CD}^{\left(1\right)},H_{CD}^{\left(3\right)}\right]+c_{2}c_{3}\left[H_{CD}^{\left(2\right)},H_{CD}^{\left(3\right)}\right]\right\}+\ldots \\ \; & =e^{-i\delta t\sum_{j=1}^{3}c_{j}H_{CD}^{\left(j\right)}+\frac{1}{2}{\left(-i\delta t\right)}^{2}\left\{c_{1}c_{2}\left[H_{CD}^{\left(1\right)},H_{CD}^{\left(2\right)}\right]+c_{1}c_{3}\left[H_{CD}^{\left(1\right)},H_{CD}^{\left(3\right)}\right]+c_{2}c_{3}\left[H_{CD}^{\left(2\right)},H_{CD}^{\left(3\right)}\right]\right\}+\ldots } \end{array}$$

## 4. Extension to the Transverse Ising Chain

The characteristic of commutative closure is not exclusive to the two-qubit system. Rather, it is applicable in a broader context within certain larger-size models. In this part, the transverse Ising chain model is identified as an appropriate framework for this fidelity-improving method. Now, we consider the time-dependent transverse Ising chain with a nearest-neighbor interaction:

(23) $$H_{0}\left(t\right)=-\left(1-\lambda \left(t\right)\right)\sum_{i}^{N}h\sigma_{i}^{x}-\lambda \left(t\right)\sum_{i}^{N}J\sigma_{i}^{z}\sigma_{i+1}^{z},$$

with the periodic boundary condition $\sigma_{N+1}^{z}=\sigma_{1}^{z}$. Here, the parameter λ follows the schedule $\lambda \left(t\right)=\sin^{2}\left[\left(\pi /2\right)\sin^{2}\left(\pi t/2T\right)\right]$. For the numerical simulation, we set J=h=1 to adopt the conventional condition of quantum annealing. To meet the exact CD driving, the nested commutators are expanded to the (N−1) order. The counter-diabatic Hamiltonian can be easily derived by consolidating the recurring operators in the expansions:

(24) $$\begin{array}{cc} H_{CD} & =\dot{\lambda }\sum_{k=1}^{N-1}{\tilde{\alpha }}_{k}\sum_{i=1}^{N}\left[\sigma_{i}^{y}{\left(\sigma_{i}^{x}\right)}^{\left(k-1\right)}\sigma_{i+k}^{z}+\sigma_{i}^{z}{\left(\sigma_{i}^{x}\right)}^{\left(k-1\right)}\sigma_{i+k}^{y}\right], \end{array}$$

where

(25) $${\left(\sigma_{i}^{x}\right)}^{\left(k-1\right)}=\prod_{j=1}^{k}\sigma_{i+j}^{x},\;k=2,\ldots ,N-1,$$

and ${\left(\sigma_{i}^{x}\right)}^{\left(0\right)}=I$. The original time-dependent coefficients, ${\tilde{\alpha }}_{k}$, can be numerically received with the approach of [31].

As the simplest simulation, we set N=3, and thus, the operator pool of CD terms includes the following: $\{\sigma_{i}^{y}\sigma_{i+1}^{z},\sigma_{i}^{z}\sigma_{i+1}^{y},\sigma_{i}^{y}\sigma_{i+1}^{x}\sigma_{i+2}^{z},\sigma_{i}^{z}\sigma_{i+1}^{x}\sigma_{i+2}^{y}\},i=1,2,3$, 12 operators in total. The corresponding coefficients can be presented only by ${\tilde{\alpha }}_{1}$ and ${\tilde{\alpha }}_{2}$. In order to utilize the transformation equations similar to (19), it is essential to remark all the coefficients. Typically, the quantity of optimization equations corresponds to the number of coefficients involved, resulting in an escalation in computational demands. However, it is possible to mitigate this increase by reorganizing and consolidating terms that incorporate the commutating operators. Here, we present a form of reorganization: $H_{CD}^{\left(0,0\right)}=\sigma_{3}^{y}\sigma_{1}^{z}+\sigma_{2}^{z}\sigma_{3}^{y}$, $H_{CD}^{\left(0,1\right)}=\sigma_{1}^{y}\sigma_{2}^{z}+\sigma_{1}^{z}\sigma_{2}^{y}$, $H_{CD}^{\left(0,2\right)}=\sigma_{3}^{z}\sigma_{1}^{y}+\sigma_{2}^{y}\sigma_{3}^{z}$, $H_{CD}^{\left(1,0\right)}=\sigma_{1}^{y}\sigma_{2}^{x}\sigma_{3}^{z}+\sigma_{3}^{z}\sigma_{1}^{x}\sigma_{2}^{y}$, $H_{CD}^{\left(1,1\right)}=\sigma_{2}^{y}\sigma_{3}^{x}\sigma_{1}^{z}+\sigma_{2}^{z}\sigma_{3}^{x}\sigma_{1}^{y}$, and $H_{CD}^{\left(1,2\right)}=\sigma_{3}^{y}\sigma_{1}^{x}\sigma_{2}^{z}+\sigma_{1}^{z}\sigma_{2}^{x}\sigma_{3}^{y}$. Consequently, there exists a correlation between these terms:

(26) $$\left[H_{CD}^{\left(p,u\right)},H_{CD}^{\left(q,v\right)}\right]=-2i\varepsilon_{uvw}\cdot H_{CD}^{\left(\delta_{pq},w\right)}.$$

**Figure 5:**
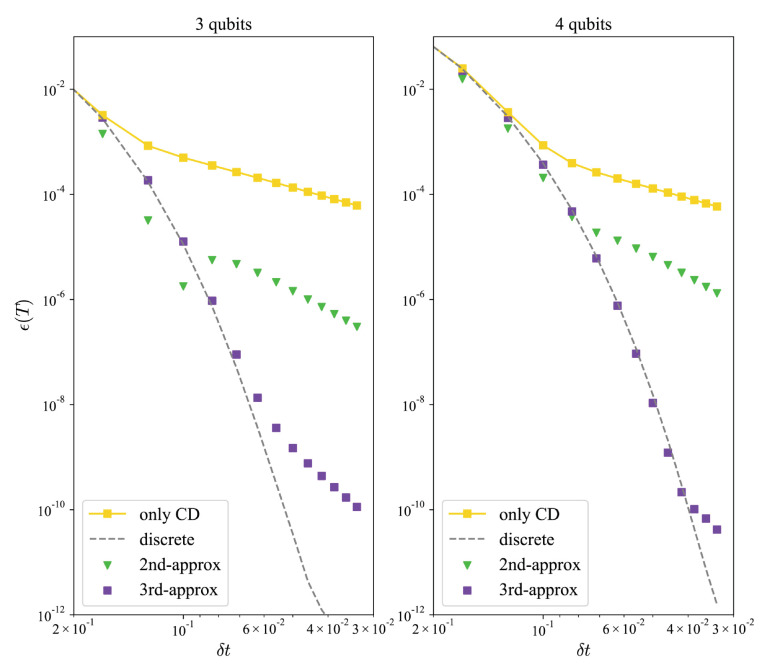
Infidelity of digitized approximate CD driving in three-qubit and four-qubit transverse Ising chain against the time slice, δt, of each step. The dashed lines are the result of the discrete effective operators, respectively. The figure verifies that the optimization of both orders of approximation improved the results. Here, parameter T=1.0.

This means a combination is allowed for each of the commutating operators $H_{CD}^{\left(0,u\right)}+H_{CD}^{\left(1,u\right)}$, and we can utilize the same method in Equation (18). The optimized time-dependent evolution operator is as follows: ${\hat{U}}_{d}^{*}\left(n\delta t\right)=\prod_{u=0}^{2}exp\left\{-i\delta t\left(a_{0,u}H_{CD}^{\left(0,u\right)}+a_{1,u}H_{CD}^{\left(1,u\right)}\right)\right\}$, where the corresponding coefficients $a_{r,u}=a_{r,u}\left(n\delta t\right)$ for r=0,1;u=0,1,2. The optimization equations can be expressed as follows:

(27) $$\left\{\begin{array}{c} a_{0,0}-\delta t\left(a_{0,1}a_{1,2}+a_{1,1}a_{0,2}\right)=\dot{\lambda }{\tilde{\alpha }}_{0}\\ a_{0,1}+\delta t\left(a_{0,0}a_{1,2}+a_{1,0}a_{0,2}\right)=\dot{\lambda }{\tilde{\alpha }}_{0}\\ a_{0,2}-\delta t\left(a_{0,0}a_{1,1}+a_{1,0}a_{0,1}\right)=\dot{\lambda }{\tilde{\alpha }}_{0}\\ a_{1,0}-\delta t\left(a_{0,1}a_{0,2}+a_{1,1}a_{1,2}\right)=\dot{\lambda }{\tilde{\alpha }}_{1}\\ a_{1,1}+\delta t\left(a_{0,0}a_{0,2}+a_{1,0}a_{1,2}\right)=\dot{\lambda }{\tilde{\alpha }}_{1}\\ a_{1,2}-\delta t\left(a_{0,0}a_{0,1}+a_{1,0}a_{1,1}\right)=\dot{\lambda }{\tilde{\alpha }}_{1} \end{array}\right.,$$

of the second-order approximation. Here, we omit the (nδt). Therefore, the total number of equations has been diminished to six. By solving the equations, the improvement in infidelity driving via the optimized ${\hat{U}}_{d}^{*}$ is shown in the left panel of Figure 5.

Furthermore, we extend the model into four qubits in order to study the capability of expansion, as shown in the right panel of Figure 5, with six remarked coefficients of the reorganized terms in the third-order CD driving. Theoretically, this optimization approach concerning coefficients is well suited to an infinite extension of the transverse Ising model due to the fact that the operators presented in Equation (24) exhibit a closure of commutative relations.

## 5. Conclusions

This work has analyzed the infidelity of digitized approximate counter-diabatic driving in terms of Trotter errors and adiabatic errors for a two-qubit system, where we can easily obtain the analytical solution for CD terms. In the l=1 CD driving, the evolution process cannot reach adiabaticity due to the incompleteness of the operator pool, resulting in infidelity mainly attributed to adiabatic errors. A suitable selection of the coefficients of the CD operators can help improve the adiabatic errors. Here, we chose the corresponding coefficients of the same operators that appear in the l=2 CD terms as an alternative, and we found an improvement of about 3.7%. We used statistical distance to find a better performance for the alternative driving in the Fubini–Study angle at each step, leading to a lower error upper bound. The l=2 CD driving achieve perfect fidelity, so Trotter errors dominate when the number of Trotter steps, *M*, is not high. By applying the BCH formula, we found a commutative closure of the operator pool in the l=2 CD terms, which results in a series of equations for optimizing the coefficients to eliminate Trotter errors. This method is also applicable to Hamiltonians whose CD terms have the same property, such as the transverse Ising chain model. A better result can be more accessible by applying this computing method, which will be helpful in gaining a deeper understanding of the dynamic behavior.

## Data Availability

The original contributions presented in the study are included in the article, further inquiries can be directed to the corresponding author.

## Appendix A. Numerical Example of Analysis on the Improvement of Fidelity

We analyzed the improvements in the first-order CD driving using Taylor expansion at one step. At any given time, nδt, the exponentiation operator $e^{-ic_{j}\left(n\delta t\right)H_{CD}^{\left(j\right)}\delta t}$ can be represented as follows:

(A1) $$e^{-ic_{j}H_{CD}^{\left(j\right)}\delta t}=I-ic_{j}H_{CD}^{\left(j\right)}\delta t+\frac{1}{2}{\left(-ic_{j}H_{CD}^{\left(j\right)}\delta t\right)}^{2}+\ldots ,$$

where j=1,2, and (nδt) is omitted for convenience, as in the following derivation.

So, Equation (16) can be represented as Equation (21), where ${\hat{U}}_{0,n}=e^{-iH_{0}\delta t}$, and $E_{0}=E_{0}\left(n\delta t\right)$ is the instantaneous ground-state energy. Here, the distance is written as a $cos(\Theta_{n})$ function. On the right side of this equation, the fidelity is divided into many parts, based on the expansion order. We keep the expansion to the second order. Since these parts are all inner products, the result of $cos(\Theta_{n})$ can be described as a sum of numbers $a_{k}+ib_{k},k=0,1,2$ with different orders of δt. Therefore, $a_{0}+ib_{0}=\langle {\hat{U}}_{0,n}^{†}{\tilde{\psi }}_{n}|{\tilde{\psi }}_{n-1}\rangle$ has no relation with CD terms. But the rest of $a_{k}+ib_{k}$ also has non-neglectable contributions, which are only dependent on the coefficients $c_{1}$ and $c_{2}$ because the corresponding inner products $\langle {\hat{U}}_{0,n}^{†}{\tilde{\psi }}_{n}|H_{CD}^{\left(j\right)}|{\tilde{\psi }}_{n}\rangle$ are fixed in one certain Trotter step. In this model, $a_{k}+ib_{k}$ are all real numbers, so $b_{k}=0,\forall k$. Here, we list the values of $a_{k}$ for n=10,M=20:

**Table A1:** Values of each part in $cos(L_{10})$.

	$a_{0}$	$\delta {\mathrm{ta}}_{1}$	$\delta t^{2}a_{2}$
original	0.99638	0.00403	−0.00140
alternative	0.99638	0.00571	−0.00260

Note that $c_{1}\left(10\delta t\right)$ undergoes little change; $a_{1}$ and $a_{2}$ are mainly affected by $c_{2}\left(10\delta t\right)$. The sum of $a_{k}$ in the alternative undergoes about a 0.0005 improvement in fidelity at step n=10, leading to a lower $L_{10}$. Generally, the lower the $L_{n}$ at each step, the shorter the statistical distance. Thus, the adiabatic errors are reduced by changing the coefficients.
